# Bioactive Components From *Gracilaria rubra* With Growth Inhibition on HCT116 Colon Cancer Cells and Anti-inflammatory Capacity in RAW 264.7 Macrophages

**DOI:** 10.3389/fnut.2022.856282

**Published:** 2022-04-06

**Authors:** Lingxiao Yi, Qi Wang, Haiyan Luo, Daqing Lei, Zhonghai Tang, Sijia Lei, Hang Xiao

**Affiliations:** ^1^Department of Food Science, University of Massachusetts, Amherst, MA, United States; ^2^School of Food and Drug, Shenzhen Polytechnic, Shenzhen, China; ^3^College of Food Science and Technology, Hunan Agricultural University, Changsha, China

**Keywords:** *Gracilaria rubra*, extractable components, non-extractable components, anti-colon cancer, anti-inflammation

## Abstract

*Gracilaria rubra* is rich in bioactive compounds with various potential health benefits. This study aimed to elucidate the profile of both extractable bioactive components (EBCs) and non-extractable bioactive components (NEBCs) of *G. rubra* and determine their anti-colon cancer and anti-inflammatory activities. Both EBCs and NEBCs displayed strong suppressive effects on the viability of HCT116 cells, which causes cell cycle arrest, induces cellular apoptosis, and regulates the expression of cyclin-dependent kinases (CDKs) and tumor suppressor proteins. Additionally, EBCs and NEBCs from *G. rubra* displayed anti-inflammatory functions *via* inhibiting the production of nitric oxide (NO), reactive oxygen species (ROS), and proinflammatory cytokines in activated macrophages and regulating the expression levels of cyclooxygenase-2 (COX-2), inducible nitric oxide synthase (iNOS), NADPH-quinone oxidoreductase-1 (NQO-1), and heme oxygenase 1 (HO-1). These findings provide a rationale for animal and human studies designed to evaluate the chemopreventive and anti-inflammatory potential of these bioactive compounds from *G. rubra*.

## Graphical Abstract

Extractable bioactive components (EBCs) and non-extractable bioactive components (NEBCs) have been isolated from *Gracilaria rubra*, which contains certain amounts of phenolics, flavonoids, tannins, anthocyanins, proteins, and carbohydrates. Additionally, EBCs and NEBCs displayed anti-colon cancer capacities and anti-inflammatory properties in cell studies.

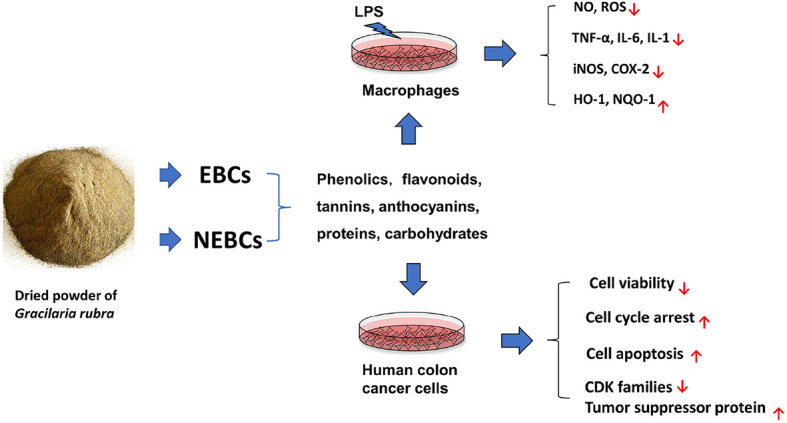

## Introduction

Colon cancer is the third most commonly diagnosed cancer in the United States (1). The etiology of colon cancer is associated with various factors that include genetics, diet, lifestyle, and the immune system. The relationship between inflammation and colon cancer has been established and supported by genetic, pharmacological, and epidemiological data (2). For instance, patients with inflammatory bowel diseases (IBDs) are at higher risk of developing colon cancer, compared with age-matched general populations (3). The current research focuses on developing alternative approaches using food components to prevent cancers and chronic inflammation. Bioactive components from plant-based food, especially whole grains, fruits, and vegetables, have been reported with protective effects against colon cancer and colonic inflammation in cell and animal studies (4–6).

Seaweeds have been served as natural bioactive substances and possess health benefits against inflammation, obesity, diabetes, cancer, and oxidative stress (7). Seaweed extracts exerted

suppressive effects on colon cancer and inflammatory agents in cell studies (8, 9). Moreover, anti-colon cancer and anti-inflammation abilities of seaweed extracts have been reported in rodent models (10, 11). The genus of *Gracilaria* is commonly found in the tropical regions that have been used in traditional Chinese medicine for obesity, cardiovascular disease, and chronic disease in the digestive system, respiratory system, urine system, and endocrine system (12). Various types of *Gracilaria* are rich in polyphenolic compounds and exert potent antioxidant capacity, which includes *G. changii* and *G. gracilis* (13, 14). Further studies found that bioactive components from the species of *Gracilaria* are reported with inhibitory effects on multiple types of cancer cells (15, 16). Recent studies reported that the sulfated polysaccharide from the *G. rubra* produced strong antioxidant and immunostimulating activities (17). However, the literature focused on the composition and biological activities for the components from *G. rubra* is rare, in comparison with other species of *Gracilaria*.

Multiple bioactive components have been quantified from *Gracilaria* with different biological properties against chronic disease (17, 18). Most of the bioactive components from *Gracilaria* are acquired by the extraction methods using organic solvents, which belong to the extractable bioactive components (EBCs). However, recent studies revealed that there were still some bioactive components that remained in the residues after extraction, such as non-extractable polyphenols, which are considered as non-extractable bioactive components (NEBCs) (19). These NEBCs may reach the colon intact and be released from the food matrix, producing small molecules which may possess health benefits (20). Until now, the literature concerning the bioactive components in *G. rubra* against inflammation and colon cancer is rare, particularly for the NEBCs from *Gracilaria*, which have been neglected. Thus, we aimed to elucidate the composition of EBCs and NEBCs from *G. rubra*, and also to investigate their potential anti-colon cancer and anti-inflammatory efficacy and mechanism in this study.

## Materials and Methods

### Materials

Fresh *G. rubra* were harvested and collected from Sandu Gulf in Fujian province, China, in the summer of 2018. The seaweed sample was washed, freeze-dried, homogenized, and stored in the freezer before use. Apigenin, luteolin, epicatechin, gallocatechin (GC), epigallocatechin (EGC), epicatechin gallate (EGCG), rutin, hesperidin, morin, quecertin, caffeic acid, vanillic acid, and rosmarinic acid were acquired from Shyuanye (Shanghai, China).

### Preparation of Bioactive Components

The dried powder of *G. rubra* was blended with 80% (v/v) chilled acetone aqueous. The blend was subjected to ultrasonic vibration for half an hour and then spin at 3,000 g for 5 min. The same procedure was repeated three times to assess residues. After that, the supernatants were collected, concentrated, and used for the isolation of EBCs, and the rest residues were used for the isolation of NEBCs.

#### Preparation of Extractable Bioactive Components

The pooled supernatants were dissolved in ethanol, followed by the addition of hexane to isolate fat and chlorophyl II. After that, the ethanol layer was collected, concentrated, and assessed by the extraction of ethyl acetate three times. Finally, the upper layer was concentrated, dried, and stored in the freezer for further analysis.

#### Preparation of Non-extractable Bioactive Components

The residues were blended with sodium hydroxide (2M) at 37°C for 2 h under a nitrogen atmosphere to avoid the oxidation of phenolic compounds before the addition of concentrated hydrochloric acid adjusted pH to 2 to terminate the reaction. The blend was spinning at 4,000 g for 5 min before the aqueous supernatant was extracted three times with ethyl-acetate. Finally, the upper layer was collected, concentrated, dried, and stored in the freezer for further analysis.

### Investigation of Total Phenolic Contents, Flavonoid Contents, Tannin Contents, Carbohydrate Contents, Reducing Sugar Contents, Anthocyanin Contents, Total Protein Contents, and Oxygen Radical Absorbance Capacity

Total phenolic contents (PCs) were assessed by Folin–Ciocalteu method with modifications, and gallic acid was used as standard (21). A volume of 20 μl of Folin–Ciocalteu reagent was mixed with 20 μl of distilled water and 20 μl of sample in a 96-well plate and stand at room temperature for 10 min, before adding 140 μl of 7% sodium carbonate solution to the plate. After incubation at room temperature for another 90 min, the absorbance was monitored at 760 nm using a spectrophotometer (BioTek Instrument, Inc., Winooski, VT, United States). PCs were calculated as μg of gallic acid equivalents per g dried powder (μg GAE/g dried powder).

Flavonoid contents (FCs) were determined using the aluminum trichloride method with modification, and catechin was used as standard (22). A volume of 20 μl of the sample was added to a 96-well plate with 10 μl of 5% sodium nitrite solution and 100 μl of distilled water, which was kept at room temperature for 6 min. Next, a volume of 20 μl of 10% aluminum chloride solution was added to the plate and reacted for 5 min, before adding 50 μl of 1 M NaOH and reacting for 2 min. The absorbance was monitored at 510 nm using a spectrophotometer (BioTek Instrument). FCs were calculated as μg of catechin equivalents per g dried powder (μg CE/g dried powder).

Tannin contents (TCs) were determined by the vanillin-sulfuric acid method with modification, and catechin was used as standard (23). The vanillin-H_2_SO_4_ solution was prepared with the same volume of 4% vanillin in methanol and 30% H_2_SO_4_ in methanol. A volume of 20 μl of the sample was mixed with 180 μl of vanillin-H_2_SO_4_ solution in a 96-well plate. The absorbance was calculated at 510 nm using a spectrophotometer (BioTek Instrument). FCs were presented as μg of catechin equivalents per g dried powder (μg CE/g dried powder).

Carbohydrate contents (CCs) were evaluated by phenol–sulfuric acid methods with modification, and glucose was used as standard (24). A volume of 50 μl of the sample was mixed with 30 μl of 5% phenol and 150 μl of concentrated sulfuric acid in a 96-well plate. After heating at 90°C for 5 min, the absorbance was monitored at 490 nm using a spectrophotometer (BioTek Instrument). CCs were calculated as μg of glucose equivalents per g dried powder (μg GE/g dried powder).

Reducing sugar contents (RSCs) were evaluated by the DNS method with minor modification, and glucose was used as standard (25). The dinitrosalicylic (DNS) solution was prepared with the 1:1:1:1 volumetric mixture of 1% 3,5-dinitrosalicylic acid, 40% Rochelle salt, 0.2% phenol, 0.5%, and potassium disulfide which was dissolved in 1.5% sodium hydroxide. A volume of 100 μl of the sample was added into 96-well plates with 100 μl of DNS solution. The plate was incubated at 90°C for 10 min before the absorbance was monitored at the wavelength of 540 nm. RSCs were presented as μg of glucose equivalents per g dried powder (μg GE/g dried powder).

Anthocyanin contents (ACs) were evaluated by the pH differential method with modification (26). A volume of 100 μl of the sample was added to the 96-well plate with two groups. A volume of 160 μl of potassium chloride buffer, pH 1.0, was added into one group, and the other with 160 μl sodium acetate buffer, pH 4.5. The absorbance was measured at 510 and 700 nm in buffers at pH 1.0 and 4.5, respectively. ACs have presented as μg cyanidin 3-glucoside equivalents per g sample.

Total protein contents (PRCs) were evaluated by the BCA method (27). The absorbance was monitored at 562 nm, and PRCs were presented as μg of protein per g dried powder (μg protein/g dried powder).

Oxygen radical absorbance capacity (ORAC) test was performed following the previously published method with modification, and Trolox was used as standard (28). A volume of 20 μl of the sample was mixed with 40 μl of 75 μM of fluorescencein solution in a 96-well plate and gently shaken at 37°C for 2 min, followed by adding 140 μl of 0.8 M of 2,2′-azobis(2-amidinopropane) dihydrochloride solution. The absorbance was measured at the excitation and emission wavelength at 485 and 528 nm, respectively. This process continued for 2 h, and the absorbance was recorded with an interval of 2 min. Results were calculated as μmol of Trolox equivalents per g dried powder (μmol TE/g dried powder).

### HPLC/MS Analysis

Phenolic compounds were quantified by Ultimate 3000 RSLC HPLC coupled with Orbitrap Fusion Mass Spectrometer (Thermo Fisher Scientific, Waltham, MA, United States). Chromatography separation was carried out by the Zorbax SB-Aq C18 column (250 mm × 4.6 mm, 5 μm, Agilent Technologies, Santa Clara, CA, United States). Meanwhile, the mobile phase is made up of 5% methanol with 0.1% formic acid (solvent A) and 0.1% formic acid in 100% methanol (solvent B). The initial mobile phase was 10% B maintained for 4 min before linearly increased to 80% within the next 11 min and maintained for 5 min. Then, the concentration of solvent B was linearly reduced to 10% within 10 min and maintained for 5 min. The flow rate was 600 μl/min, and the injection volume was 20 μl. Data were acquired in negative ESI mode using a spray voltage of 3,000 V, with sheath and aux gas set to 40 and 10, respectively, and also vaporizer and tube temperature set to 300 and 275°C. Data processing was accomplished using Xcalibur V4.1 (Thermo Fisher Scientific, Waltham, MA, United States).

### Cell Viability Assay

Assay of cell viability was monitored as we reported previously (19). CCD-18Co (10,000 cells/well), HCT116 cells (2,500 cells/well), and RAW 264.7 (1,00,000 cells/well) were cultured in 96-well plate and incubated overnight, before posed to multiple concentrations of EBCs or NEBCs. CCD-18Co cells were assessed to MTT assay after 72 h of treatment. HCT116 cells were assessed to MTT assay after 24, 48, and 72 h of treatment, respectively. RAW 264.7 macrophages were carried out to MTT assay after 24 h of treatment.

### Flow Cytometry Analysis

Flow cytometry analysis was carried out as we reported previously described with minor modifications (29). HCT116 cells (4 × 10^4^ cells/ml) were cultured in 6-well plates and incubated overnight before posed to EBCs or NEBCs with 24 h for cell cycle analysis and 48 h for cell apoptosis analysis. Subsequently, media containing any floating cells were collected by trypsinization. Finally, cell pellets were washed by chilled PBS and subject flow cytometry as we reported previously (29).

### Determination of Nitric Oxide and Reactive Oxygen Species Production

Griess reactions were carried out to evaluate NO production as previously reported (30). RAW264.7 cells (1 × 10^5^ cells/well) were cultured into 96-well plates and incubated overnight before posed to multiple concentrations EBCs or NEBCs with 1 μg/ml lipopolysaccharides (LPS) for another 24 h. Next, culture media were assessed to Griess reaction.

Reactive oxygen species (ROS) production was measured according to the previous report with minor modifications (31). RAW264.7 cells were cultured in 96-well plates and incubated overnight before being processed by multiple concentrations of EBCs or NEBCs with 1 μg/ml LPS for 24 h. Then, the cells were assessed to a dichlorofluorescein-diacetate (DCFH-DA) assay.

### Real-Time qRT-PCR Analysis

Real-time qRT-PCR analysis was carried out as we reported previously (30). The primer pairs were used for the cDNA amplification and are listed in Supplementary Table 1. Three independent parallel groups were carried out, and related messenger RNA (mRNA) expression was evaluated by the 2^–ΔΔCt^ method (32).

### Immunoblotting Analysis

The whole-cell extract preparation was based on the previous report (30). The RIPA buffer containing protease inhibitor cocktail and phosphatase inhibitor (Boston BioProducts, Ashland, MA, United States) was used to lyse cells. The antibodies of iNOS, HO-1, NQO-1 COX-2, p53, and p21 were ordered from Santa Cruz Biotechnology (Dallas, TX, United States), and antibodies of CDK2, CDK4, and CDK6 were ordered from Cell Signaling Technology (Beverly, MA, United States). The antibody of β-actin was ordered from Sigma-Aldrich.

### Statistical Analysis

Data were presented as mean ± standard derivation (SD) of more than three independent experiments. Analysis of variance (ANOVA) and Student’s *t*-test were performed to compare the difference between two or more groups. *P*-Value < 0.05 was considered statistically significant.

## Results and Discussion

### Composition of Extractable Bioactive Components and Non-extractable Bioactive Components

**FIGURE 1:**
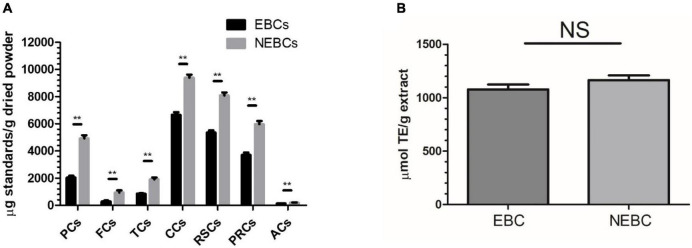
**(A)** The total phenolics contents (PCs), flavonoids contents (FCs), tannin contents (TCs), carbohydrate contents (CCs), reducing sugar contents (RSCs), anthocyanin contents (ACs), total protein contents (PRCs) in the EBCs and NEBCs. **(B)** The ORAC value of the EBCs and NEBCs. Data were presented as mean ± SD (*n* = 3), ** indicates *P* < 0.01.

In this study, acidic acetone was used to isolate EBCs from *G. rubra*, and alkaline hydrolysis was performed to release NEBCs from the matrix of *G. rubra*. The yields of EBCs and NEBCs were 1,200 and 2,700 mg per 100 g dried powder, respectively. The total contents of PCs, FCs TCs, CCs, RSCs, PRCs, and ACs are shown in Figure 1. There were certain amounts of phenolics, flavonoids, tannins, carbohydrates, reducing sugar, and protein in the EBCs and NEBCs. In contrast, fewer amounts of anthocyanins and flavonoids were detected in EBCs and NEBCs. The amounts of PCs, FCs, TCs, CCs, RSCs, and PRCs in EBCs were lower than that in NEBCs. Few anthocyanins (less than 1 mg cyanidin 3-glucoside equivalents per g dry powder) were detected in EBCs and NEBCs. The ORAC was carried out to evaluate the antioxidant capacities of EBCs and NEBCs. The ORAC values of EBCs and NEBCs were 1,077.35 and 1,164.94 μmol TE/g (dried powder), respectively.

High-resolution MS was performed to analyze the polyphenol compounds, which are secondary plant metabolites with health benefits against different types of cancer, inflammation, cardiovascular disease, obesity, and diabetes (33). Rutin, morin, quercetin, caffeic acid, vanillic acid, EGCG, epicatechin, hesperidin dominated in EBCs, and few GC, ECG, apigenin, luteolin, and rosmarinic acid were identified (Table 1). The previous studies reported that rutin, morin, caffeic acid, ECG, EGCG, GC, hesperidin, eckol, phlorotannins were the major phenolic compounds identified in various types of seaweeds extracted by organic solvent (34). In NEBCs, the identified phenolic compounds contained rutin, epicatechin, morin, quercetin, and smaller amounts of EGCG, hesperidin, luteolin, rosmarinic acid, and apigenin (Table 1). Alkaloid hydrolysis was reported to release bioactive compounds such as polyphenols in the residues *via* breaking the ester and glycoside linkage (35). Herein, our results first clarified the chemical profiles of bioactive compounds in the non-extractable parts of the *Gracilaria*. NEBCs may be released by breaking down the covalent bonds, hydrogen bonds, and/or hydrophobic interaction linked to fiber, protein, and carbohydrate (36). The different composition between the EBCs and NEBCs could influence their biological properties. Next, we aimed to understand the protective effects of these novel *G. rubra* extracts against colon cancer and inflammation.

**TABLE 1:** Phenolic compounds quantified in extractable bioactive components (EBCs) and non-extractable bioactive components (NEBCs) from *Gracilaria rubra.*

Compound	Retention time (min)	MS (m/z)	EBC (μ g/g extract)	NEBC (μ g/g extract)
Rutin	17.19	609.145 [M-H]^–^	347.84 ± 42.12	267.74 ± 29.87
Morin	18.62	301.034 [M-H]^–^	72.49 ± 8.54	17.74 ± 2.26
Epicatechin	13.78	289.071 [M-H]^–^	295.21 ± 32.29	113.96 ± 14.07
Hesperidin	17.00	609.181 [M-H]^–^	218.74 ± 27.22	78.86 ± 8.16
EGCG	14.74	457.077 [M-H]^–^	128.78 ± 14.62	16.12 ± 1.88
Caffeic acid	15.39	179.034 [M-H]^–^	100.39 ± 12.81	78.86 ± 8.16
Quecertin	19.05	301.034 [M-H]^–^	19.14 ± 4.67	102.47 ± 12.28
Vanillic acid	15.57	167.034 [M-H]^–^	38.45 ± 4.62	ND
GC	13.19	305.066 [M-H]^–^	12.08 ± 2.52	ND
ECG	15.58	441.082 [M-H]^–^	3.71 ± 0.53	ND
Rosmarinic acid	17.09	359.076 [M-H]^–^	3.73 ± 0.58	0.65 ± 0.10
Luteolin	19.85	285.039 [M-H]^–^	ND	0.57 ± 0.09
Apigenin	20.57	269.045 [M-H] ^–^	1.45 ± 0.24	0.96 ± 0.18

*Results were presented as mean ± SD; ND, not detected.*

### Extractable Bioactive Components and Non-extractable Bioactive Components Lowered the Viability of Colon Cancer Cells

**FIGURE 2:**
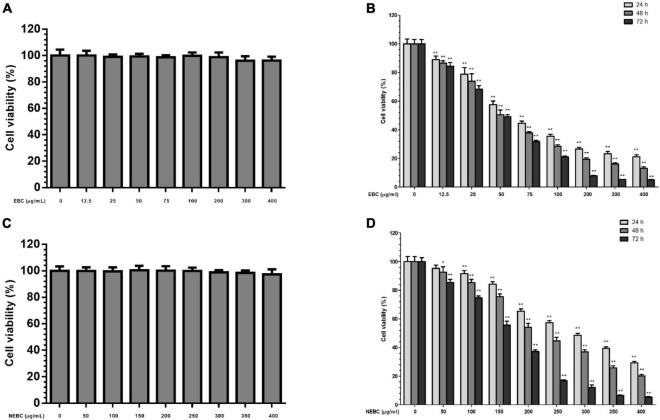
Effects of EBCs **(A)** and NEBCs **(C)** on the growth of CCD18-Co cells for 72 h; Inhibitory effects of EBCs **(B)** and NEBCs **(D)** on the growth of HCT116 cells for 24, 48, and 72 h. * indicates *P* < 0.05 and ** indicates *P* < 0.01, when compared to the untreated control group.

MTT assay was carried out to monitor the suppressive properties of EBCs and NEBCs on the normal colon cells and colon cancer cells, respectively. As shown in Figure 2, EBCs and NEBCs did not display cytotoxicity effects on the growth of CCD18-Co cells up to 400 μg/ml for 72 h (Figures 2A,C). Moreover, EBCs and NEBCs slightly lowered the cell viability of HCT116 cells. Accurately, the half-maximal inhibitory concentration (IC_50_) values of EBCs were 71.40, 53.50, and 43.90 μg/ml at 24, 48, and 72 h, respectively (Figure 2B), and the IC_50_ values of NEBCs were 283.7, 226.9, and 153.4 μg/ml at 24, 48, and 72 h, respectively (Figure 2D). Overview, our results illustrated that EBCs and NEBCs potently reduced the viability of colon cancer cells, whereas there were no cytotoxicity effects on the normal colon cells at higher concentrations. Furthermore, EBCs displayed stronger inhibitory effects on the growth of colon cancer cells, compared with the NEBCs. Thus, we selected EBCs, at 50 and 100 μg/ml, and NEBCs, at 200 and 300 μg/ml, according to the results of the MTT assay, for the further mechanistic test.

### Extractable Bioactive Components and Non-extractable Bioactive Components Led Cell Cycle Arrest and Apoptosis in HCT116 Cells

**FIGURE 3:**
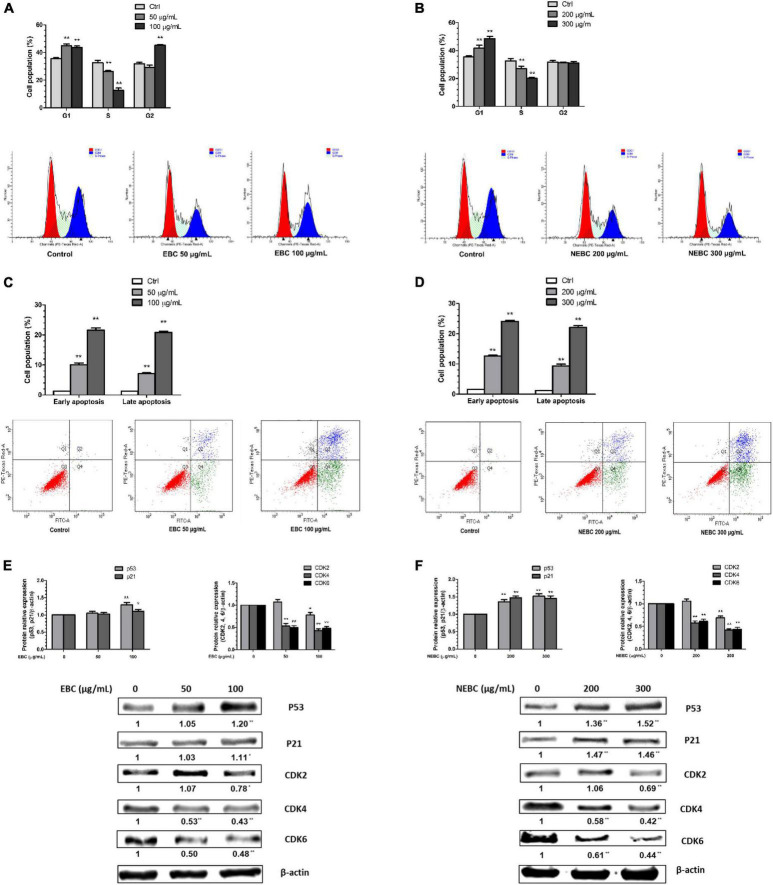
Quantification of cell cycle after treatment of EBCs **(A)** and NEBCs **(B)** and the representative DNA histogram. Quantification of early and late apoptosis after treatment of EBCs **(C)** and NEBCs **(D)** and the representative Annexin V/PI co-staining dot plots. Effects of EBCs **(E)** and NEBCs **(F)** on the protein expression of tumor suppress genes and cyclin-dependent kinase in HCT 116 cells, as well as their representative image. * indicates *P* < 0.05 and ** indicates *P* < 0.01, when compared to the untreated control group.

Flow cytometry analysis was carried out to explore the molecular mechanism by which EBCs and NEBCs lowered the viability of colon cancer cells. Cell cycle analysis showed that both EBCs and NEBCs slightly elevated the cell percentage in the G0/G1 phase, which indicates a cell cycle arrest at G0/G1 phase (Figures 3A,B). EBCs at 50 and 100 μg/ml increased the cell percentage in the G0/G1 phase by 1.26- and 1.23-fold, respectively, whereas NEBCs at 200 and 300 μg/ml increased the cell percentage by 1.17- and 1.37-fold, respectively. Moreover, EBCs at 100 μg/ml increased the cell accumulation in the G2/M phase by 1.45-fold (Figures 3A,B).

Cell apoptosis analysis revealed that both EBCs and NEBCs significantly elevated the apoptotic cell populations. EBCs at 100 μg/ml potently induced late and early apoptotic cell populations by 16.53- and 16.87-fold, respectively. NEBCs at 300 μg/ml also markedly induced late and early apoptotic cell populations by 18.23- and 15.64-fold, respectively (Figures 3C,D). These results indicated that both EBCs and NEBCs lowered colon cancer cell growth *via* inducing cellular apoptosis and causing cell cycle arrest.

### Extractable Bioactive Components and Non-extractable Bioactive Components Modified Cell Cycle and Apoptosis-Related Proteins

Tumor suppressor genes, such as p53 and p21, regulate cellular apoptosis and cell cycle (37). We found that both EBCs and NEBCs enhanced the expression of p53 and p21. EBCs at 100 μg/ml elevated the protein expression of p53 and p21 by 20 and 11%, respectively. Similarly, NEBCs at 300 μg/ml elevated the protein expression levels of p21 and p53 by 462 and 52%, respectively (Figures 3E,F). Moreover, cyclin-dependent kinase (CDK) involves regulating the cell cycle (37). We also found that both EBCs and NEBCs lowered the expression of the CDK family. Specifically, EBCs at 100 μg/ml suppressed the expression of CDK2, CDK4, and CDK6 by 22, 57, and 52%, respectively. NEBCs at 300 μg/ml suppressed the expression levels of CDK2, CDK4, and CDK6 by 31, 58, and 64%, respectively (Figures 3E,F). Overall, these results indicated that EBCs and NEBCs from *G. rubra* displayed anti-colon cancer properties by regulating the expression of multiple tumor suppresser genes and CDKs.

### Inhibition of Extractable Bioactive Components and Non-extractable Bioactive Components on the Production of Nitric Oxide, Reactive Oxygen Species, and Proinflammatory Cytokines

**FIGURE 4:**
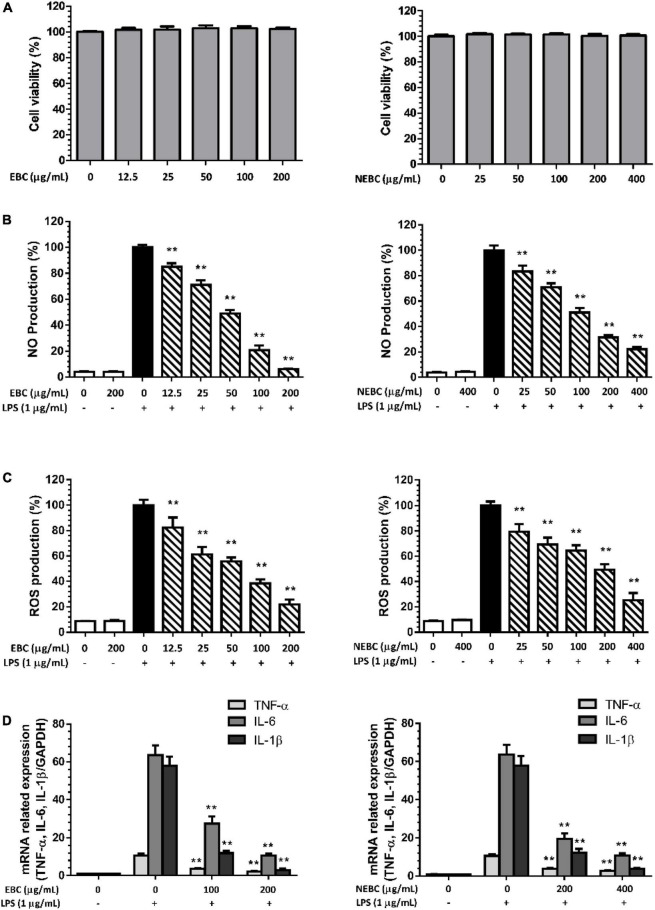
Cytotoxicity of EBCs and NEBCs in macrophages **(A)**. Suppressive effects of EBCs and NEBCs on NO **(B)** and ROS **(C)** production in activated macrophages. Inhibitory effects of EBCs and NEBCs in messenger RNA (mRNA) expression level of tumor necrosis factor alpha (TNF-α), Interleukin (IL)-6 and IL-1β in activated macrophages **(D)**. ** indicates *P* < 0.01 in comparison with the LPS group.

In this study, macrophages activated by LPS were carried out to measure the anti-inflammation properties of EBCs and NEBCs. First, we monitored the cytotoxicity effects in macrophages up to 200 μg/ml for EBCs and up to 400 μg/ml for NEBCs, based on the MTT assay. We found that neither EBCs nor NEBCs showed cytotoxicity in the range aforementioned (Figure 4A). As shown in Figure 4B, LPS stimulation alone remarkably elevated the production of NO. However, EBCs and NEBCs reduced the LPS-induced NO production in a dose-dependent manner. More accurately, the IC_50_ values of EBCs and NEBCs were 44.81 and 107.05 μg/ml, respectively. The highest concentration of EBCs (200 μg/ml) and NEBCs (400 μg/ml) significantly reduced the NO production by 93.9 and 77.9%, respectively. LPS can induce cell oxidative damage *via* promoting ROS generation (31). As shown in Figure 4C, LPS stimulation alone greatly enhanced the production of ROS, whereas EBC and NEBC treatments lowered the ROS production in a dose-dependent manner. Specifically, the IC_50_ values of EBCs were 58.33 μg/ml, which is lower than the NEBCs at 148.52 μg/ml. EBCs at 200 μg/ml and NEBCs at 400 μg/ml significantly reduced the ROS production by 78.0 and 75.0%, respectively. These results indicated that EBCs and NEBCs exerted anti-inflammatory capacities *via* reducing the production of NO and ROS in activated macrophages. Moreover, EBCs displayed stronger anti-inflammatory capacities than NEBCs.

Furthermore, LPS stimulation significantly raised mRNA expression of tumor necrosis factor alpha (TNF-α), Interleukin (IL)-6, and IL-1β by 10. 72-, 60. 56-, and 53.04-fold compared with the control group, respectively. EBCs and NEBCs reduced the raised expression of these cytokines in a dose-dependent manner (Figure 4D). EBCs at 200 μg/ml and NEBCs at 400 μg/ml greatly lowered mRNA expression of TNF-α by 79 and 73%, respectively. Similar patterns were obtained in the mRNA expression of IL-6 and IL-1β. Collectively, our results illustrated that EBCs and NEBCs exerted an anti-inflammatory role *via* attenuating the production of NO and ROS and downregulating expression levels of proinflammatory cytokines in activated macrophages.

### Regulation of Inflammation-Related Signaling Proteins by Extractable Bioactive Components and Non-extractable Bioactive Components in Macrophages

**FIGURE 5:**
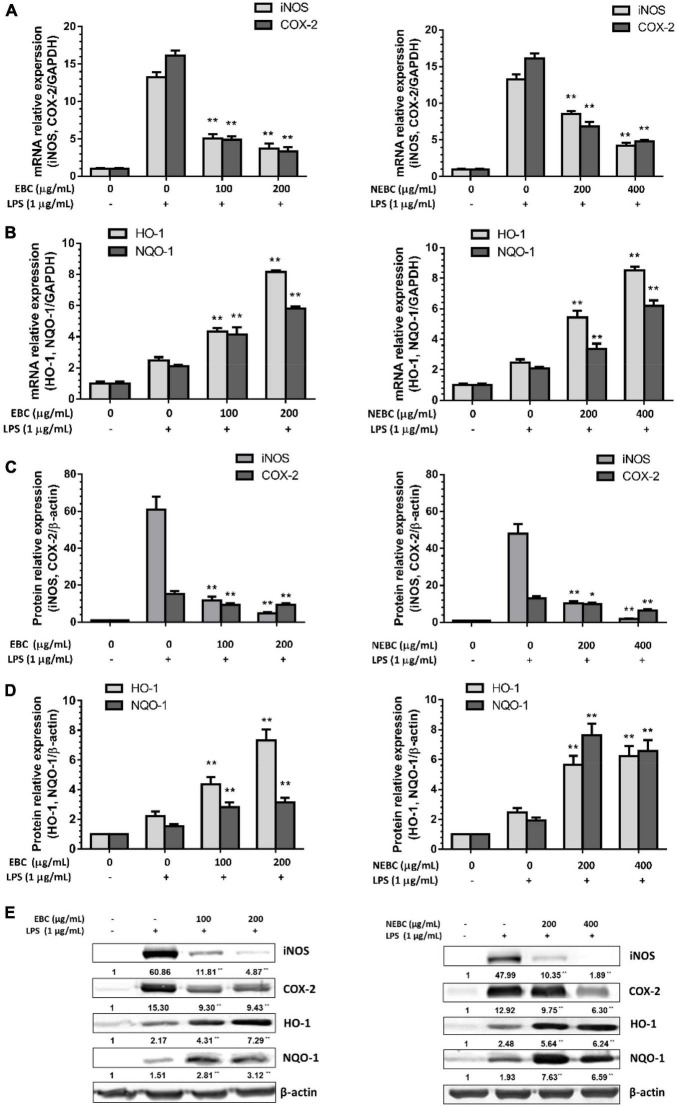
Suppressive effects of EBCs and NEBCs on mRNA **(A)** expression and protein **(C)** expression levels of iNOS and COX-2 in activated macrophages. Effects of EBCs and NEBCs on mRNA **(B)** and protein **(D)** expression levels of HO-1 and NQO-1 in activated macrophages. Representative image of immunoblotting for the effects of EBCs and NEBCs on protein expression levels of iNOS, COX-2, HO-1 and NQO-1 in activated macrophages **(E)**. * indicates (*P* < 0.05) and ** indicates (*P* < 0.01), when compared to the LPS group.

Activated macrophages induce the overproduction of proinflammatory enzymes, namely, cyclooxygenase-2 (COX-2) and inducible nitric oxide synthase (iNOS) (38, 39). Compared with the control group, LPS stimulation significantly raised the expression of mRNA levels of iNOS by 13.26-fold. EBCs at 200 μg/ml significantly reduced the mRNA levels of iNOS by 72% and NEBCs at 400 μg/ml diminished iNOS expression levels by 67%, compared with the LPS group (Figure 5A). Additionally, LPS stimulation alone greatly elevated the protein levels of iNOS by 57.0-fold. EBCs at 200 μg/ml and NEBCs at 400 μg/ml markedly lowered the protein levels of iNOS by 91 and 96%, compared with the LPS group (Figures 5C,E). Similar results were found in the analysis of mRNA and protein levels of COX-2 (Figures 5A,C,E). Our results suggested that EBCs and NEBCs attenuated the production of NO-induced by LPS *via* downregulating the iNOS and COX-2 signaling pathways.

Anti-oxidative enzymes, especially for HO-1 and NQO-1, are linked to anti-inflammatory functions (40). We found that EBCs at 200 μg/ml elevated the mRNA levels of HO-1 by 3.29-fold and NEBCs at 400 μg/ml elevated the mRNA levels of HO-1 by 2.35-fold, respectively, compared with LPS groups. Additionally, EBCs and NEBCs also enhanced mRNA expression levels NQO-1 (Figure 5B). The protein levels of HO-1 and NQO-1 were consistent with mRNA expression levels. EBCs at 200 μg/ml raised protein levels of HO-1 and NQO-1 by 2.18- and 1.89-fold, respectively, compared with the LPS group. NEBCs at 400 μg/ml increased the levels of HO-1 and NQO-1 by 3.66- and 5.79-fold, respectively (Figures 5D,E). Our results indicated that the upregulation of anti-oxidative enzymes HO-1 and NQO-1 by EBCs and NEBCs from *G. rubra* might repair the damage caused by oxidative stress in inflammatory diseases.

## Conclusion

Our studies offered an incisive understanding of the composition and biological properties of different bioactive components present in *G. rubra*. These results, for the first time, evaluated the biological function of bioactive components in *G. rubra* against colon cancer and inflammation agent, especially for the NEBCs that are always ignored in the current research concerning the seaweed bioactive components. These components in NEBCs normally reach the colon intact, where they are released from the food matrix due to the fermentation by gut microbiota. EBCs and NEBCs from *G. rubra* contained varying amounts of phenolics, flavonoids, tannins, carbohydrates, and proteins and showed potent antioxidant capacities. Our research demonstrated that both EBCs and NEBCs from *G. rubra* exert potent biological activities against colon cancer and inflammation. Accurately, EBCs displayed robust inhibitory effects suppressing the growth of colon cancer cells and strong anti-inflammatory capacities in activated macrophages. Further studies indicated that EBCs and NEBCs suppressed the growth of HCT116 colon cancer cells *via* causing cellular apoptosis and cell cycle arrest. Also, EBCs and NEBCs displayed their anti-inflammatory function *via* downregulating the expression levels of NO, ROS, proinflammatory cytokines, and enzymes and upregulating the expression levels of antioxidant enzymes.

## Data Availability Statement

The original contributions presented in the study are included in the article/Supplementary Materials, further inquiries can be directed to the corresponding author/s.

## Author Contributions

LY: methodology, experiment performance, data collection, analysis, and writing—original draft preparation. QW: data collection and analysis. HL: sample preparation. DL: manuscript revision. ZT: supervision. SL: conceptualization and supervision. HX: conceptualization, supervision, writing reviewing, and editing. All authors contributed to the article and approved the submitted version.

## Conflict of Interest

The authors declare that the research was conducted in the absence of any commercial or financial relationships that could be construed as a potential conflict of interest.

## Publisher’s Note

All claims expressed in this article are solely those of the authors and do not necessarily represent those of their affiliated organizations, or those of the publisher, the editors and the reviewers. Any product that may be evaluated in this article, or claim that may be made by its manufacturer, is not guaranteed or endorsed by the publisher.

## Abbreviations

EBCs: extractable bioactive components
NEBCs: non-extractable bioactive components
LPS: lipopolysaccharides
NO: nitrite oxide
ROS: reactive oxygen species
iNOS: inducible nitric oxide synthase
COX-2: cyclooxygenase-2
HO-1: heme oxygenase 1
NQO-1: NADPH-quinone oxidoreductase-1
TNF-α: tumor necrosis factors-α
IL: interleukin
PCs: phenolics contents
FCs: flavonoid contents
TCs: tannin contents
CCs: carbohydrate contents
RSCs: reduced sugar contents
ACs: anthocyanin contents
PRCs: protein contents
ORAC: oxygen radical absorbance capacity
CDK: cyclin-dependent kinase.
