# Supplementary material for: Bioactive Components From Gracilaria rubra With Growth Inhibition on HCT116 Colon Cancer Cells and Anti-inflammatory Capacity in RAW 264.7 Macrophages
Source: Front Nutr. 2022 Apr 6;9:856282. doi: 10.3389/fnut.2022.856282 (PMC9019662; doi:10.3389/fnut.2022.856282)
Supplement: Supplementary file 1 [file Table_1.docx]

**Supplementary information**

Table.S1 Primers pairs used for cDNA amplification

| Primer | Direction | Sequence | Source |
| --- | --- | --- | --- |
| iNOS | Forward  Reverse | 5′-TCC TAC ACC ACA CCA AAC-3′  5′-CTC CAA TCT CTG CCT ATC C-3′ | ([1](#_ENREF_1)) |
| COX-2 | Forward  Reverse | 5′-CCT CTG CGA TGC TCT TCC-3′  5′-TCA CAC TTATAC TGG TCA AAT CC-3′ |  |
| HO-1 | Forward  Reverse | 5′-AAG AGG CTA AGA CCG CCT TC-3′  5′-GTC GTC GTC AGT CAA CAT GG-3′ |  |
| NQO-1 | Forward  Reverse | 5′-TCG GAG AAC TTT CAG TAC CC-3′  5′-TGC AGA GAG TAC ATG GAG CC-3’ |  |
| GADPH | Forward  Reverse | 5′-TCA ACG GCA CAG TCA AGG-3′  5′-ACT CCA CGA CAT ACT CAG C-3′ |  |
| TNF-α | Forward  Reverse | 5′-ATG AGC ACA GAA AGC ATG ATC-3’  5′-TAC AGG CTT GTC ACT CGA ATT-3′ | ([2](#_ENREF_2)) |
| IL-1β | Forward  Reverse | 5′-ATG GCA ACT GTT CCT GAA CTC AAC T-3′  5′-TTT CCT TTC TTA GAT ATG GAC AGG AC-3′ |  |
| IL-6 | Forward  Reverse | 5′-AGT TGC CTT CTT GGG ACT GA-3′  5′-CAG AAT TGC CAT TGC ACA AC-3′ |  |

1. Wu X, Song M, Rakariyatham K, Zheng J, Guo S, Tang Z, et al. Anti-inflammatory effects of 4'-demethylnobiletin, a major metabolite of nobiletin. *J Funct Foods* (2015) 19(Pt A):278-87. doi: 10.1016/j.jff.2015.09.035. PubMed PMID: 26770275; PubMed Central PMCID: PMC4707668.

2. Ryu B, Choi I-W, Qian Z-J, Heo S-J, Kang D-H, Oh C, et al. Anti-inflammatory effect of polyphenol-rich extract from the red alga Callophyllis japonica in lipopolysaccharide-induced RAW 264.7 macrophages. *Algae* (2014) 29(4):343-53. doi: 10.4490/algae.2014.29.4.343.
